# Impacts of specific acupuncture techniques combined with infrared radiation therapy on the symptom improvement and quality of life of patients with allergic rhinitis

**DOI:** 10.3389/fmed.2026.1805445

**Published:** 2026-04-29

**Authors:** Yan Fan, Hongchao Xing

**Affiliations:** Department of Geriatric Rehabilitation, Jincheng Rehabilitation Hospital, Jincheng, Shanxi, China

**Keywords:** acupuncture, allergic rhinitis, Dazhu (BL11), IgE, infrared irradiation, Tongtian (BL7)

## Abstract

**Background:**

Allergic rhinitis (AR) is a prevalent chronic inflammatory condition with significant global health and socioeconomic burdens. Conventional pharmacotherapy often has limitations, driving interest in complementary approaches like acupuncture. However, heterogeneous protocols hinder reproducibility. This study evaluated a standardized, innovative acupuncture protocol integrating strong manual stimulation at specific acupoints with infrared irradiation for AR.

**Methods:**

In this randomized, assessor-blinded, controlled trial, 120 patients were equally separated into a study group or a control group. The former adopted oral loratadine for 14 days. The latter received a 14-session intervention combining acupuncture at Tongtian (BL7) and Dazhu (BL11) with infrared irradiation. Primary outcomes included the Total Nasal Symptom Score (TNSS), Total Non-Nasal Symptom Score (TNNSS), as well as a Visual Analogue Scale (VAS). Secondary outcomes were the Rhinoconjunctivitis Quality of Life Questionnaire (RQLQ) score, serum IgE levels, eosinophil (EOS) counts, serum levels of IL-4, IL-6 as well as IFN-*γ* as well as adverse events.

**Results:**

Relative to the control group, the study group demonstrated significantly greater reductions in TNSS, TNNSS, and VAS scores at all post-treatment and follow-up assessments (mid-treatment, end-of-treatment, 1-month, and 3-month follow-up; all *p* < 0.05). Improvements in RQLQ scores were also significantly greater in the study group at all time points (all *p* < 0.05). Furthermore, the study group showed more pronounced decreases in serum IgE levels, peripheral blood EOS counts as well as serum levels of IL-4, IL-6 and IFN-*γ* (all *p* < 0.05). The incidence of adverse reactions (e.g., drowsiness, dry mouth) presented significantly lower in the study group (*p* < 0.05).

**Conclusion:**

A standardized acupuncture protocol combining strong stimulation at BL7 and BL11 with infrared irradiation is significantly more effective and safer than loratadine monotherapy for managing AR. It provides superior and sustained relief of nasal and systemic symptoms, greater improvement in quality of life, more effective modulation of allergic-immunological markers and inflammatory factors, and a better safety profile. This integrated approach represents a promising non-pharmacological treatment option.

## Introduction

Allergic rhinitis (AR) belongs to a chronic inflammatory disease that occurs in the nasal mucosa (1). Its main clinical manifestations include paroxysmal sneezing, copious clear nasal discharge, nasal itching as well as nasal congestion (2). AR is a global health concern with a substantial and growing prevalence, posing significant socioeconomic burdens due to its impact on productivity, healthcare costs, as well as quality of life (3). In China, the rising incidence of AR aligns with broader environmental and lifestyle changes, making it a pressing public health issue (4). Conventional Western treatments—primarily pharmacotherapy (e.g., antihistamines, corticosteroids) and allergen immunotherapy—offer symptomatic relief but are often limited by side effects, variable efficacy, and poor long-term adherence (5). As a result, complementary and alternative therapies, particularly traditional Chinese medicine (TCM) treatment, have gained increasing clinical acceptance and are increasingly recommended in guidelines as adjunctive or alternative options for AR management (6).

AR, known in TCM as *Biqiu*. The term *Biqiu* has been documented since ancient classical texts such as the *Yellow Emperor’s Inner Classic*, reflecting its long-standing recognition in East Asian medical systems (7). According to TCM theory, the pathogenesis of *Biqiu* primarily involves deficiency of the Lung, Spleen, or Kidney, combined with invasion of external pathogenic wind. The Lung governs respiration and defensive qi; deficiency may lead to impaired resistance against environmental pathogens. The Spleen is responsible for transformation and transportation; its deficiency can result in damp accumulation and phlegm formation. The Kidney, as the foundation of the body’s yang and root of qi, when deficient, fails to anchor qi and warm the upper respiratory pathways. These internal deficiencies, when coupled with external factors containing wind-cold or wind-heat, disrupt the normal function of the nose, leading to the hypersensitivity and inflammatory responses characteristic of AR (8).

TCM treatment for *Biqiu* typically includes herbal medicine, moxibustion, Tuina, and acupuncture, all aimed at strengthening the body’s defensive capacity, dispelling pathogenic factors, and regulating organ function (9). Among these, acupuncture is widely utilized for its capacity to modulate qi and blood, harmonize yin and yang, and regulate the function of the lung and related meridians (10). Acupuncture has demonstrated promise in alleviating AR symptoms and improving patient-reported outcomes (11). However, its application remains heterogeneous, with inconsistencies in point selection, stimulation parameters, and treatment protocols, which hinder reproducibility and mechanistic understanding. Furthermore, while existing research supports the general efficacy of acupuncture for AR, studies focusing on specific point combinations, intensified manipulation techniques, and synergistic integration with physical modalities remain scarce.

This study addresses key gaps by proposing a standardized protocol combining specific acupuncture points with defined manual techniques and infrared irradiation. The selected points— “Tongtian” (BL7) and “Dazhu” (BL11)—are grounded in classical theory: *Tongtian* is indicated for nasal obstruction and connects directly with the nasal orifice, while *Dazhu*, the influential point of bone, governs the body’s surface and aligns with the theory that “the lung is associated with the skin and body hair.” Together, these points are theorized to strengthen the exterior, dispel wind, and regulate the lung’s diffusing function.

Thus, the primary objective of this research is to evaluate the clinical efficacy and safety of a standardized acupuncture protocol combining strong stimulation at Tongtian (BL7) and Dazhu (BL11) with infrared irradiation in patients with AR.

## Methods

### Study design and participants

This study adopted a randomized, controlled, single-blind (assessor-blinded) trial design. From March 2024 to March 2025, 120 patients with AR who were treated in our hospital were selected as the research subjects. This study was approved by the hospital’s ethics committee, and all patients signed the informed consent form. Ethics approval number is JC2024_L030025, and the document has been provided as Supplementary File 1. Trial registration number is ChiCTR2200061597.

Inclusion Criteria: (1) Age between 18 and 65 years; (2) Patients met the international criteria outlined in the “Allergic Rhinitis and Its Impact on Asthma” (ARIA) guidelines (12), which meant they have persistent moderate to severe allergic rhinitis; and (3) Stable medication regimen for AR (if any) for at least 4 weeks prior to enrollment, with agreement to maintain it unchanged during the study period unless required for rescue.

Exclusion criteria: (1) Severe systemic diseases (e.g., cardiovascular, hepatic, renal, hematopoietic disorders); (2) Nasal surgery within the past 6 months or significant anatomical nasal deformity; (3) Pregnant or lactating women; (4) Participation in another clinical trial within the past 3 months; and (5) Contraindications to acupuncture (e.g., bleeding disorders, needle phobia) or infrared therapy (e.g., local skin infections, impaired thermal sensation).

Sample size calculation was performed *a priori* using GPower software with the following parameters: Cohen’s d = 0.72 (calculated as mean difference/pooled SD); *α* (two-sided) = 0.05; Power (1−*β*) = 0.80; Allocation ratio = 1:1.

### Randomization and allocation concealment

Utilizing a computer-generated randomization sequence by SAS 9.4, patients were allocated to either the control group or the study group on a 1:1 basis. Permuted block randomization with variable block sizes (4, 6, and 8) was employed to ensure balanced group allocation. The randomization sequence was created by an independent statistician who had no involvement in patient recruitment, administration of interventions, or outcome evaluation. Central randomization was not used due to the single-center design. However, allocation concealment was strictly maintained using sequentially numbered, opaque, sealed envelopes. The treating clinician opened the envelope only after the participant had completed all baseline assessments.

To maintain allocation concealment and minimize selection bias, the group allocations were enclosed in sequentially numbered, opaque, and sealed envelopes. Each envelope was marked solely with a distinct study identification number. Following the acquisition of written informed consent and the completion of baseline evaluations, the clinician responsible for treatment opened the envelope that matched the participant’s study number to disclose the group assignment. This approach ensured that the allocation remained concealed until the moment of intervention. A CONSORT flow diagram was provided in Supplementary Figure 1A.

### Methods

The control group was given oral loratadine tablets, 10 mg per tablet, 1 tablet each time, once a day, before bedtime. A 7-day course was considered as one treatment session. The treatment was carried out continuously for 2 courses, totaling 14 sessions.

The study group was given acupuncture combined with infrared therapy.

Acupoint selection:

Main points: Bilateral Tongtian (BL7) and Dazhu (BL11).

Adjunctive points: Fengchi (GB20) and Kongzui (LU6) may be added based on TCM pattern differentiation.

Operational specifications:

Tongtian (BL7): A sterile disposable acupuncture needle (0.30 mm × 40 mm) was inserted subcutaneously at an angle of 15–20 degrees to a depth of 0.5–0.8 inches. Upon insertion, a strong stimulation technique involving vigorous lifting, thrusting, and rotating manipulations was applied to elicit a pronounced *deqi* sensation (characterized as soreness, numbness, distension, or a radiating sensation), ideally directed towards the nasal and forehead region, within the patient’s tolerance.

Dazhu (BL11): A sterile disposable acupuncture needle (0.30 mm × 40 mm) was inserted perpendicularly to a depth of 0.5–0.8 inches. The “thread-lifting technique” (*Zhi Zhen* technique) was employed, which involved rotating the needle unidirectionally (e.g., clockwise) until a distinct tightening sensation, indicative of the needle being grasped by surrounding tissue, was achieved. This was combined with periodic strong manual stimulation. An illustration of Tongtian and Dazhu was provided in Supplementary Figure 1B.

Infrared Therapy: Concurrently with the needling at Dazhu (BL11), a standardized infrared therapy lamp (Model: CQJ-22A Infrared therapy lamp; wavelength: 0.8–1.5 μm; output power: 250 W) was positioned vertically at a fixed distance of 30 cm above the skin surface over the bilateral Dazhu acupoint area. The irradiation was applied continuously throughout the 30-min needle retention period. The skin temperature at the irradiation site was monitored to ensure it remained within a safe and therapeutic range (typically 40–43 °C).

Needle retention and stimulation: All needles were retained for 30 min. During this period, manual stimulation (repeating the described techniques) was performed for approximately 30 s at each acupoint every 10 min.

Treatment course: Once a day. Each 7 sessions constitute one treatment course. The treatment was carried out continuously for 2 courses, totaling 14 sessions. The trial schedule was provided in Table 1.

**Table 1:** Schedule of enrollment, interventions, and assessments.

Time point	Enrollment	Allocation	Treatment phase	Mid-treatment	End of treatment	Follow-up
	Week 1	Week 0	Weeks 1–2	Week 1 (Day 7)	Week 2 (Day 14)	Month 1, Month 3
Enrollment						
Eligibility screen	√					
Informed consent	√					
Baseline assessment	√					
Allocation		√				
Interventions						
Control group (loratadine)			14 days			
Study group (acupuncture + infrared)			14 sessions (daily)			
Assessments						
TNSS, TNNSS, VAS, RQLQ	√			√	√	√
Blood sampling (IgE, EOS, cytokines)	√				√	√
Adverse events monitoring			Continuously			√

All patients were followed up for 3 months after completion of the treatment protocol. Follow-up assessments were conducted at 1 month and 3 months post-treatment via outpatient clinic visits. During the follow-up period, patients were instructed to maintain their usual lifestyle and avoid initiating any new treatments for AR without prior consultation. Rescue medication (oral levocetirizine 5 mg, maximum one dose per 24 h) was permitted for severe symptom exacerbation that significantly impaired daily functioning. Patients were required to record any rescue medication use in a diary, including date, time, and reason for use. The number of rescue medication doses used during the follow-up period was documented and compared between groups as an additional exploratory outcome. Patients who required rescue medication on more than three consecutive days or for more than 7 total days during the follow-up period were considered treatment failures and were managed according to clinical need, with their last observation carried forward for analysis.

Missing data were minimal in this study due to the short intervention period and close follow-up. All 120 enrolled patients completed the full treatment protocol and all scheduled assessments. Therefore, no imputation methods were required for the primary analysis, and a complete-case analysis was performed.

### Primary outcomes

The total nasal symptom score (TNSS) was implemented for assessing the nasal symptom (13). This scale includes four symptoms: nasal itching, nasal congestion, runny nose as well as sneezing. All the symptoms are rated on a scale of 5 levels based on their severity. The total TNSS score is the sum of all the symptom scores. The higher the score, the more severe the nasal symptoms.

The total non-nasal symptom score (TNNSS) was implemented for assessing the nasal associated symptoms (14). This scale includes symptoms such as nasal discharge flowing into the throat, tearing, eye itching, pain in the nose or upper part of the mouth, and headache, each scored 1 point (with symptoms) or 0 points (without symptoms). The higher the score, the more severe the nasal associated symptoms.

The Visual Analogue Scale (VAS) was implemented for assessing the degree of symptoms (15), containing nasal congestion, runny nose, sneezing, nasal itching, and reduced sense of smell. The symptom scores ranged from 0 to 10, with 0 meaning no symptoms and 10 meaning the most severe symptoms that were unbearable.

### Secondary outcomes

The quality of life of patients was evaluated utilizing the Rhinoconjunctivitis Quality of Life Questionnaire (RQLQ) (16). This questionnaire includes seven aspects, such as daily activities, sleep conditions, nasal symptoms, non-nasal/eye symptoms, and eye symptoms. Each option is scored from 0 to 6 based on the degree of symptom distress. The higher the total score, the more severe the impact of AR on the quality of life of the patients.

A total of 3 mL of fasting venous blood were obtained from the patient, and then centrifuged at 4000 revolutions per minute for 10 min to separate the serum. The total IgE level in the serum was measured using an automatic biochemical analyzer. The number of eosinophils (EOS) in the peripheral blood of the patients was detected using a blood analyzer (XN-20 model, Sysmex). The levels of IL-4, IL-6 as well as IFN-*γ* were detected utilizing enzyme linked immunosorbent assay.

The incidence of adverse reactions, including subcutaneous hematoma, drowsiness, dry mouth and dyspepsia were recorded.

### Statistical analysis

SPSS 20.0 was used for statistical analysis. Categorical data were exhibited as frequency and percentage, and the χ^2^ test or Fisher’s exact test was used for group comparisons. Measurement data that conformed to a normal distribution were exhibited as mean ± standard deviation, and *t*-test was implemented to compare the difference between both groups. Repeated measurement analysis of variance (ANOVA) was implemented to compare the measurement data at multiple time points. Statistical significance was set at *p* < 0.05.

## Results

### Baseline data

No significant differences were seen in baseline data (gender, age and course of disease) between both groups (*p* > 0.05, Table 2).

**Table 2:** Baseline data between the two groups.

Baseline data	Control group (*n* = 60)	Study group (*n* = 60)	χ^2^/t	*P*	Z/95% CI
Gender			0.133	0.714	0.3654
Male	32 (53.33)	30 (50.00)			
Female	28 (46.67)	30 (50.00)			
Age (years)	38.90 ± 4.43	39.26 ± 4.58	0.437	0.662	−1.269 ~ 1.989
Course of disease (years)	5.30 ± 0.71	5.36 ± 0.74	0.453	0.651	−0.2022 ~ 0.3222

### Nasal symptom

Before initiating the treatment, no differences were seen in TNSS scores between both groups (*p* > 0.05). A significant reduction in TNSS was observed in both groups at the mid-treatment stage (after 7 sessions), at the conclusion of treatment (after 14 sessions), as well as during the follow-up assessments conducted 1 month and 3 months post-treatment (*p* < 0.05). Moreover, at each of these specified time points, the study group consistently exhibited lower TNSS scores compared to the control group (*p* < 0.05, 95%CI: −1.708 ~ −0.2079, Figure 1).

**Figure 1:**
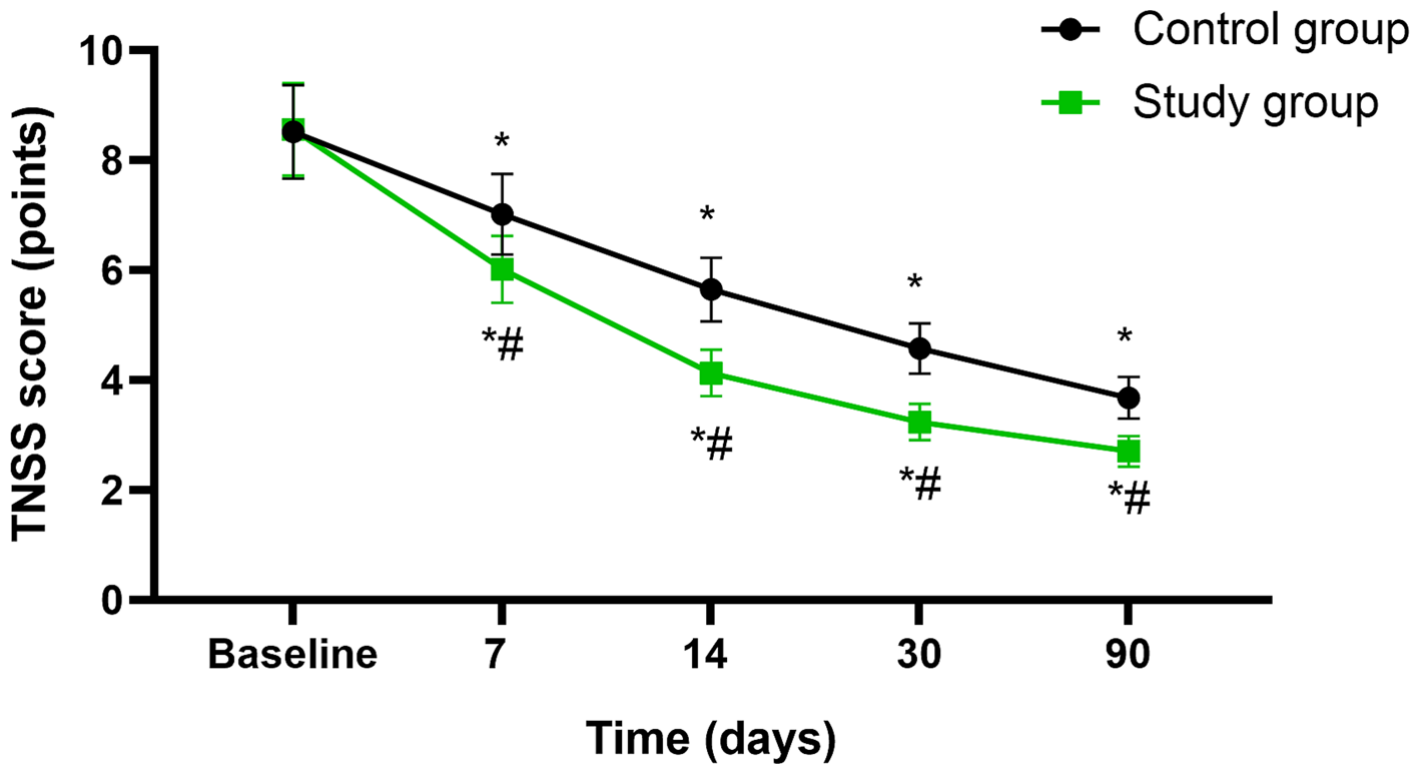
Nasal symptom between the two groups at different time points. * *p* < 0.05, compared with baseline; # *p* < 0.05, compared with control group.

### Nasal associated symptoms

**Figure 2:**
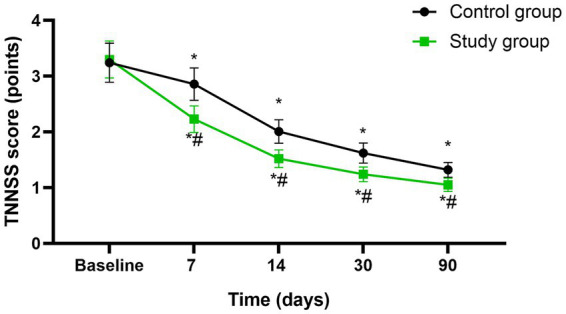
Nasal associated symptoms between the two groups at different time points. * *p* < 0.05, compared with baseline; # *p* < 0.05, compared with control group.

Before initiating the treatment, no differences were seen in TNNSS scores between both groups (*p* > 0.05). A significant reduction in TNNSS was observed in both groups at the mid-treatment stage (after 7 sessions), at the conclusion of treatment (after 14 sessions), as well as during the follow-up assessments conducted 1 month and 3 months post-treatment (*p* < 0.05), Moreover, at each of these specified time points, the study group consistently exhibited lower TNNSS scores compared to the control group (*p* < 0.05, 95%CI: −0.6664 ~ −0.01757, Figure 2).

### Degree of symptoms

**Figure 3:**
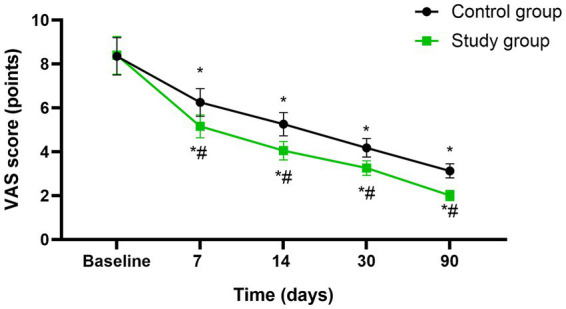
Degree of symptoms between the two groups at different time points. * *p* < 0.05, compared with baseline; # *p* < 0.05, compared with control group.

Before initiating the treatment, no differences were seen in VAS scores between both groups (*p* > 0.05). A significant reduction in VAS was observed in both groups at the mid-treatment stage (after 7 sessions), at the conclusion of treatment (after 14 sessions), as well as during the follow-up assessments conducted 1 month and 3 months post-treatment (*p* < 0.05), Moreover, at each of these specified time points, the study group consistently exhibited lower VAS scores compared to the control group (*p* < 0.05, 95%CI: −1.502 ~ −0.2144, Figure 3).

### Quality of life

**Figure 4:**
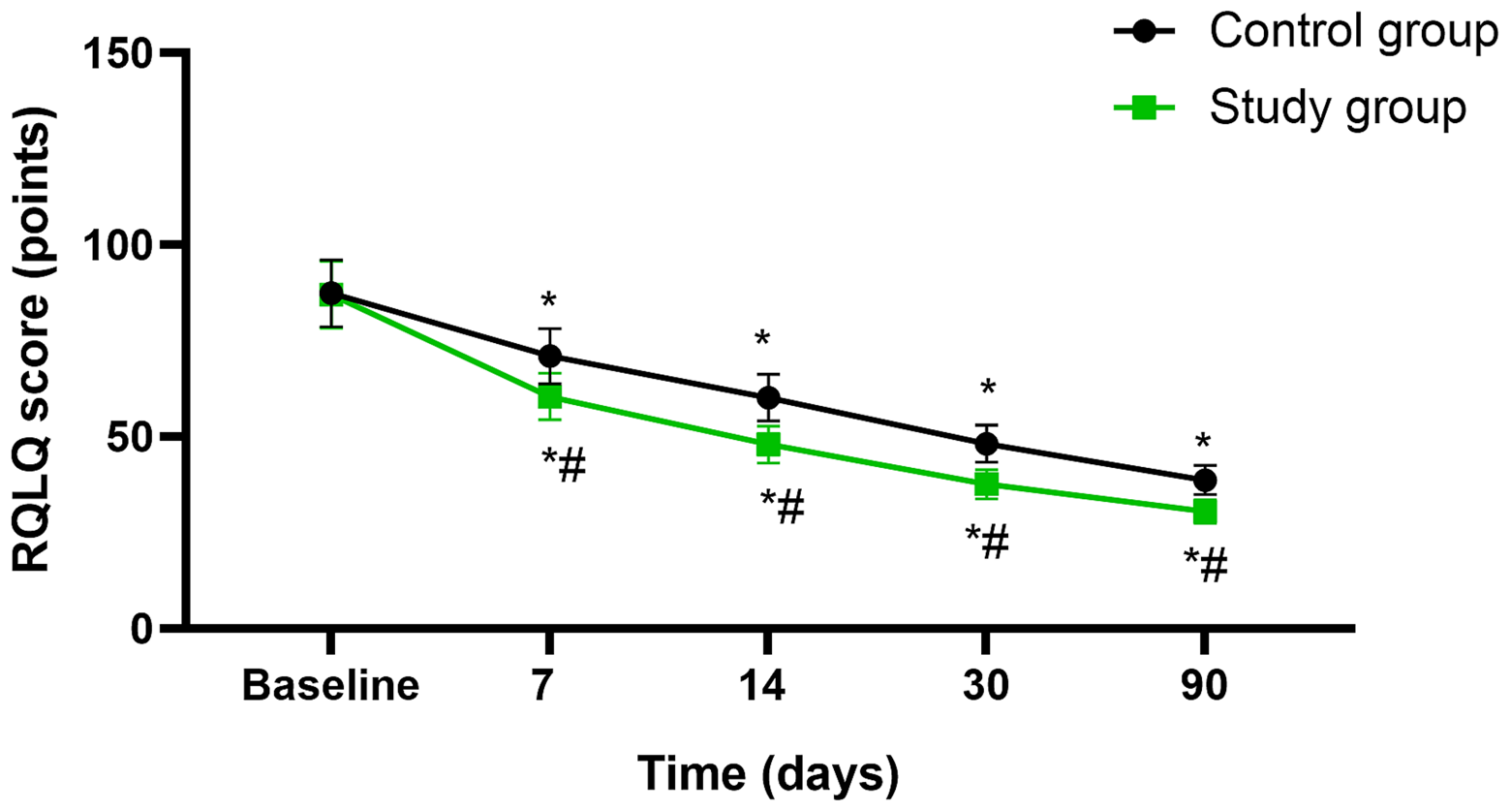
Quality of life between the two groups at different time points. * *p* < 0.05, compared with baseline; # *p* < 0.05, compared with control group.

Before initiating the treatment, no differences were seen in RQLQ scores between both groups (*p* > 0.05). A significant reduction in RQLQ was observed in both groups at the mid-treatment stage (after 7 sessions), at the conclusion of treatment (after 14 sessions), as well as during the follow-up assessments conducted 1 month and 3 months post-treatment (*p* < 0.05), Moreover, at each of these specified time points, the study group consistently exhibited lower RQLQ scores compared to the control group (*p* < 0.05, 95%CI: −14.19 ~ −2.550, Figure 4).

### Serum IgE levels and EOS counts

**Figure 5:**
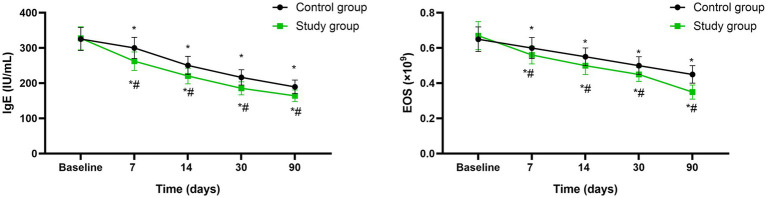
Serum IgE levels and EOS counts between the two groups at different time points. * *p* < 0.05, compared with baseline; # *p* < 0.05, compared with control group.

Before initiating the treatment, no differences were seen in serum IgE levels and EOS counts between both groups (*p* > 0.05). A significant reduction in serum IgE levels and EOS counts was observed in both groups at the mid-treatment stage (after 7 sessions), at the conclusion of treatment (after 14 sessions), as well as during the follow-up assessments conducted 1 month and 3 months post-treatment (*p* < 0.05), Moreover, at each of these specified time points, the study group consistently exhibited lower serum IgE levels (95%CI: −43.71 ~ −5.298) and EOS counts (95%CI: −0.09712 ~ 0.009117) compared to the control group (*p* < 0.05, Figure 5).

### Serum levels of IL-4, IL-6 as well as IFN-*γ*

**Figure 6:**
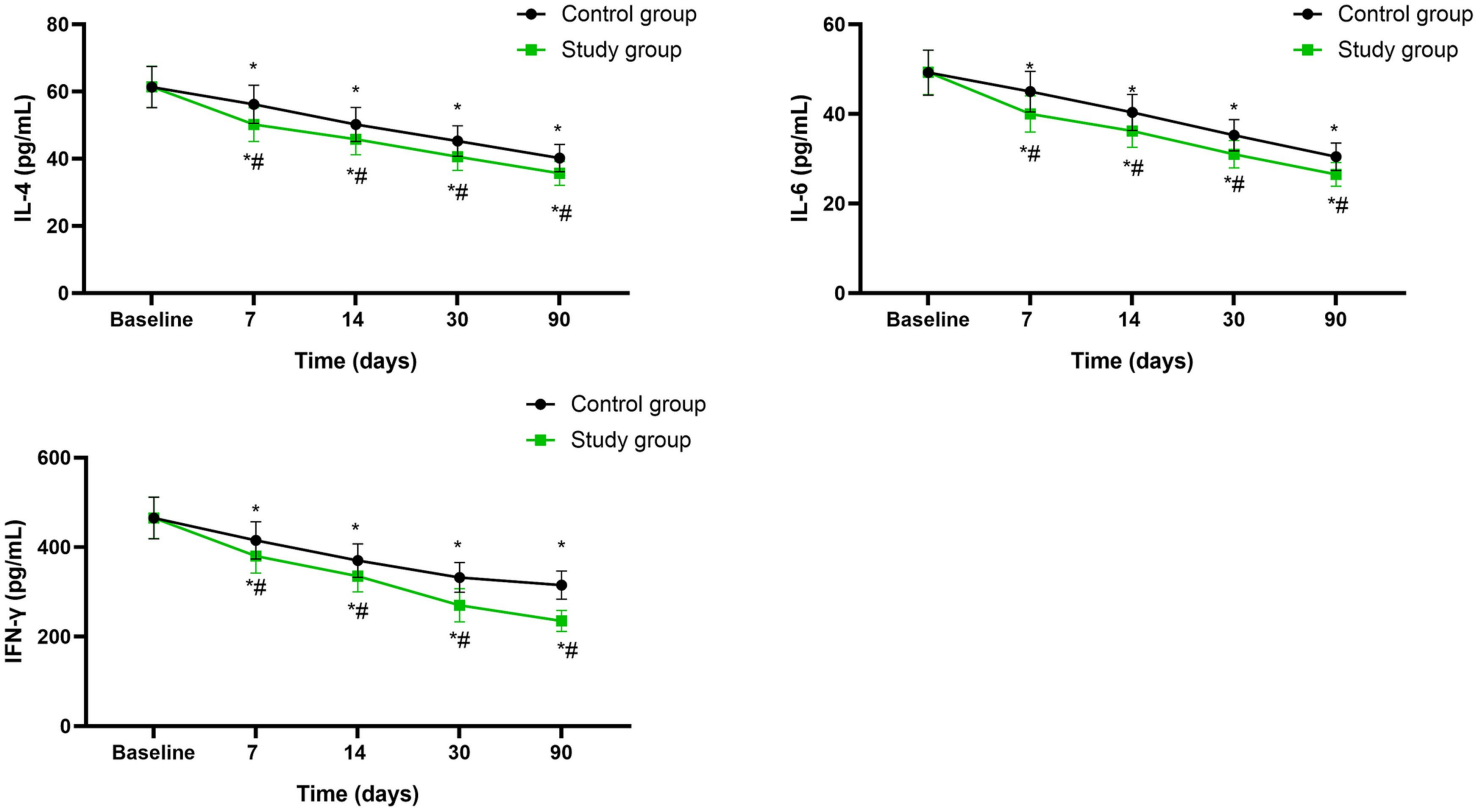
Serum levels of IL-4, IL-6 as well as IFN-*γ* between the two groups at different time points. * *p* < 0.05, compared with baseline; # *p* < 0.05, compared with control group.

Before initiating the treatment, no differences were seen in serum levels of IL-4, IL-6 as well as IFN-γ between both groups (*p* > 0.05). A significant reduction in serum levels of IL-4 (95%CI: −6.776 ~ −1.036), IL-6 (95%CI: −5.936 ~ −0.9558) as well as IFN-*γ* (95%CI: −80.07 ~ −4.620) was observed in both groups at the mid-treatment stage (after 7 sessions), at the conclusion of treatment (after 14 sessions), as well as during the follow-up assessments conducted 1 month and 3 months post-treatment (*p* < 0.05), Moreover, at each of these specified time points, the study group consistently exhibited lower serum levels of IL-4, IL-6 as well as IFN-γ compared to the control group (*p* < 0.05, Figure 6).

### Incidence of adverse reactions

As shown in Table 3, the incidence of adverse reactions in the study group was lower than that of the control group (*p* < 0.05).

**Table 3:** Incidence of adverse reactions.

Groups	Cases	Subcutaneous hematoma	Drowsiness	Dry mouth	Dyspepsia	Total incidence rate
Control group	60	0 (0.00)	2 (3.33)	3 (5.00)	3 (5.00)	8 (13.33)
Study group	60	1 (1.67)	0 (0.00)	0 (0.00)	0 (0.00)	1 (1.67)
χ^2^	4.324					
*P*	0.037					
df	3					

## Discussion

This study demonstrated that a standardized acupuncture protocol combining strong manual stimulation at Tongtian (BL7) and Dazhu (BL11) with concurrent infrared irradiation is a highly effective and safe intervention for patients with AR. Compared to oral loratadine monotherapy, the combination protocol showed statistically superior and clinically meaningful improvements across multiple domains. It led to significantly greater and more rapid reductions in core nasal symptoms (TNSS), associated non-nasal symptoms (TNNSS), and overall symptom burden (VAS), with benefits evident as early as the mid-treatment assessment (after 7 sessions) and sustained throughout the 3-month follow-up period. Furthermore, the intervention was associated with more pronounced improvements in disease-specific quality of life (RQLQ) and greater reductions in key immunological biomarkers (serum IgE and eosinophil counts) and inflammatory factors (IL-4, IL-6, IFN-*γ*), alongside a significantly lower incidence of adverse reactions. These robust and multi-dimensional results suggest that our integrated approach not only provides superior symptomatic control but also may modulate the underlying immunological dysregulation characteristic of AR.

The rapid onset and superior efficacy observed may be related to the synergistic design of the protocol, which targets AR pathophysiology from multiple angles. From a TCM perspective, the protocol induces immediate neuro-modulatory and local vascular effects. The strong manual stimulation at Tongtian (BL7), located on the forehead directly above the nasal sinuses, is theorized to regulate local anatomical structures and cranial blood supply. By eliciting a propagated *deqi* sensation toward the nasal and forehead regions, it may directly inhibit nociceptive signaling, enhance parasympathetic tone to reduce glandular secretion, and improve microcirculation in the nasal mucosa, leading to rapid relief of congestion and rhinorrhea (17). However, the precise physiological mechanisms underlying these effects require further investigation.

Secondly, the protocol aims for deep systemic regulation. The combination of the “thread-lifting” (Zhi Zhen) technique and sustained infrared irradiation at Dazhu (BL11) may contribute to the observed therapeutic effects. While the classical TCM interpretation involves regulation of yang qi and strengthening of the body’s exterior (18, 19), from a biomedical perspective, this acupoint’s location overlying the spinous processes raises the possibility of influencing paravertebral autonomic structures and potentially affecting systemic immune regulation through neuro-immune pathways (20). However, the precise mechanisms remain to be elucidated and require further investigation using advanced neuroimaging and immunological techniques. This aligns with our findings of a more significant downregulation of serum IgE and eosinophil counts compared to the control group.

The comprehensive benefits may be partially mediated by the integrated effect on the neuro-immune-endocrine network. The intense acupoint stimulation sends robust afferent signals via somatic sensory nerves to the spinal cord and brain, activating central pain-modulatory and autonomic nervous system pathways (21). This neural signaling can subsequently influence immune function by modulating the release of neuropeptides (e.g., substance P, VIP) and systemic stress hormones, which in turn regulate the activity of mast cells, eosinophils, and Th2/Th1 cytokine balance (e.g., IL-4, IL-6, IFN-*γ*) (22). While classical Th1/Th2 paradigm suggests that IFN-γ (Th1) should increase to suppress Th2 responses, allergic inflammation is more complex. In persistent moderate-to-severe AR, chronic inflammation can lead to a mixed Th1/Th2 profile with elevated levels of both Th2 (IL-4, IL-6) and Th1 (IFN-γ) cytokines. Elevated IFN-γ in this context may reflect ongoing inflammatory activity rather than protective Th1 immunity. Therefore, the reduction in all three cytokines in our study group suggests a broad suppression of overall inflammatory activity rather than a simple Th1/Th2 shift. This interpretation aligns with our finding of reduced eosinophil counts and IgE levels, indicating global attenuation of allergic inflammation. The added infrared-induced local hyperthermia may further enhance this process by increasing local blood flow, accelerating metabolic clearance of inflammatory mediators, and potentially inducing mild heat-shock protein responses that have immunomodulatory effects (23). Thus, our protocol potentially may help disrupt the cycle of immune hypersensitivity and inflammation that characterizes AR.

The primary innovation of this study lies in the strategic integration of a traditional reinforcing needling technique with a modern physical therapy modality to amplify the classical TCM principle of “warming, unblocking, and securing the exterior”. The standardized application of the strong “thread-lifting” technique at Dazhu (BL11) ensures a consistent, high-intensity stimulus aimed at eliciting a therapeutic response. When combined with targeted infrared irradiation, this approach is theorized in TCM theory to create a “warming and unblocking” effect at a strategically selected acupoint, which may enhance its regulatory influence on immune function. This combination moves beyond conventional acupuncture or isolated infrared therapy, representing a reproducible multimodal multimodal approach.

### Strengths, limitations, and future directions

The strengths of this study include its randomized controlled design, standardized and theory-guided protocol, comprehensive outcome assessment using both patient-reported and objective immunological measures, and evaluation of short to medium-term sustained effects. However, several limitations must be acknowledged. The single-center design and specific patient population may limit generalizability. The single-blind (assessor-blinded) design, though methodologically sound, cannot eliminate potential placebo effects inherent to acupuncture studies, and the lack of a sham acupuncture control group makes it difficult to completely isolate the specific impacts of needling from the overall therapeutic context. Furthermore, the 3-month follow-up period, while valuable, is not sufficient to assess long-term durability as well as disease-modifying potential.

Future research should employ multi-center designs with larger sample sizes and incorporate rigorous sham-controlled or multi-arm designs (e.g., comparing acupuncture alone, infrared alone, and the combination) to delineate the specific contribution of each component. Mechanistic studies utilizing advanced neuroimaging and detailed cytokine/transcriptomic profiling are warranted to further elucidate the biological pathways involved. Additionally, studies with longer follow-up periods (e.g., 12 months) are required to evaluate the potential for sustained remission and reduced relapse rates.

## Conclusion

In conclusion, the integrated acupuncture protocol combining strong stimulation at Tongtian (BL7) and Dazhu (BL11) with infrared irradiation demonstrates rapid, significant, and sustained superiority over conventional antihistamine therapy in alleviating symptoms, improving quality of life, modulating immune biomarkers and inflammatory factors, and reducing adverse events in patients with persistent moderate-to-severe AR. Its efficacy may be explained by a synergistic mechanism involving local neuro-vascular modulation, deep systemic regulation of defensive yang qi and potential osteoimmune functions, ultimately mediated through the neuro-immune-endocrine network. This study provides high-quality evidence supporting this novel, standardized combination as a promising non-pharmacological treatment option for AR, warranting further investigation and clinical consideration.

## Data Availability

The datasets presented in this study can be found in online repositories. The names of the repository/repositories and accession number(s) can be found in the article/Supplementary material.

## Supplementary material

SUPPLEMENTARY FIGURE 1 **(A)** CONSORT flow diagram. **(B)** An illustration of the acupoint locations (Tongtian and Dazhu).
