# Supplementary figures and images for: Impacts of specific acupuncture techniques combined with infrared radiation therapy on the symptom improvement and quality of life of patients with allergic rhinitis
Source: Front Med (Lausanne). 2026 Apr 29;13:1805445. doi: 10.3389/fmed.2026.1805445 (PMC13168131; doi:10.3389/fmed.2026.1805445)

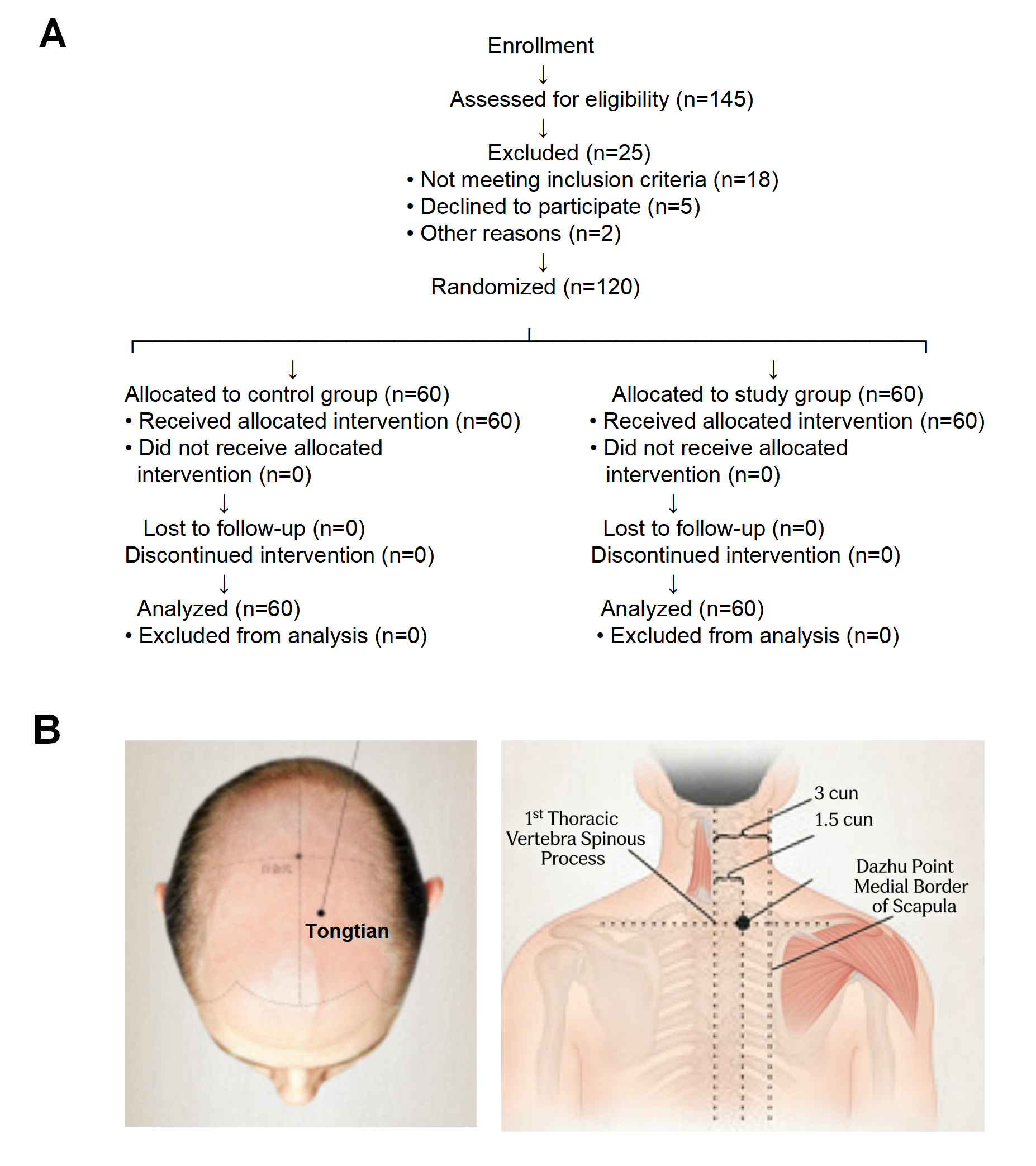

Supplement: SUPPLEMENTARY FIGURE 1 — (A) CONSORT flow diagram. (B) An illustration of the acupoint locations (Tongtian and Dazhu). [file Image_1.tif]
